# Cost savings associated with improving appropriate and reducing inappropriate preventive care: cost-consequences analysis

**DOI:** 10.1186/1472-6963-5-20

**Published:** 2005-03-09

**Authors:** William Hogg, Neill Baskerville, Jacques Lemelin

**Affiliations:** 1 Department of Family Medicine, University of Ottawa, Canada; 2 Department of Health Studies and Gerontology, University of Waterloo, Canada

## Abstract

**Background:**

Outreach facilitation has been proven successful in improving the adoption of clinical preventive care guidelines in primary care practice. The net costs and savings of delivering such an intensive intervention need to be understood. We wanted to estimate the proportion of a facilitation intervention cost that is offset and the potential for savings by reducing inappropriate screening tests and increasing appropriate screening tests in 22 intervention primary care practices affecting a population of 90,283 patients.

**Methods:**

A cost-consequences analysis of one successful outreach facilitation intervention was done, taking into account the estimated cost savings to the health system of reducing five inappropriate tests and increasing seven appropriate tests. Multiple data sources were used to calculate costs and cost savings to the government. The cost of the intervention and costs of performing appropriate testing were calculated. Costs averted were calculated by multiplying the number of tests not performed as a result of the intervention. Further downstream cost savings were determined by calculating the direct costs associated with the number of false positive test follow-ups avoided. Treatment costs averted as a result of increasing appropriate testing were similarly calculated.

**Results:**

The total cost of the intervention over 12 months was $238,388 and the cost of increasing the delivery of appropriate care was $192,912 for a total cost of $431,300. The savings from reduction in inappropriate testing were $148,568 and from avoiding treatment costs as a result of appropriate testing were $455,464 for a total savings of $604,032. On a yearly basis the net cost saving to the government is $191,733 per year (2003 $Can) equating to $3,687 per physician or $63,911 per facilitator, an estimated return on intervention investment and delivery of appropriate preventive care of 40%.

**Conclusion:**

Outreach facilitation is more expensive but more effective than other attempts to modify primary care practice and all of its costs can be offset through the reduction of inappropriate testing and increasing appropriate testing. Our calculations are based on conservative assumptions. The potential for savings is likely considerably higher.

## Background

A randomized, controlled field trial of a multifaceted intervention to improve preventive care tailored to the needs of participating family practices was conducted in Southern Ontario and delivered by nurses trained in the facilitation of prevention. This report is a cost-consequences analysis of the intervention. Specifically, it provides information about the cost of the outreach facilitator intervention and money saved the health care system as a result of reducing inappropriate and increasing appropriate preventive screening tests.

Improving preventive performance is both important and necessary. There is substantial room to improve rates of appropriate preventive practice [1]. The Canadian Task Force on the Periodic Health Examination [2,3] has established guidelines for the delivery of preventive care that are supported by clinical evidence as effective in decreasing the impact of disease. However, evidence-based guidelines are not self-implementing [4-6]. Changing physicians' long-held patterns of behaviour and the environments in which they work is essential yet complex and difficult. Unless the barriers to change can be overcome and actions taken to put preventive care guidelines into practice, evidence-based guideline development efforts will be wasted and the quality of preventive care will not improve [7].

Several reviews have focussed on the effectiveness of different interventions for implementing guidelines and improving care [5,6,8-12]. Multi-faceted outreach facilitation interventions employing trained individuals who meet with providers in their practice setting to provide information and assist the practice in implementing evidence-based guidelines have been shown to be more effective than single interventions such as guideline dissemination efforts or physician prompts [10-14]. Tailoring interventions to the requirements of the practice has also been proposed as important in supporting practice changes and in attaining more successful and sustained outcomes in preventive care performance as compared to interventions that are fixed and lack this flexibility [15-19]. Given the diversity of practice environments, it is unlikely that "one size fits all" approaches to improving preventive care will be able to address the needs of all providers and their patients [20,21].

Successful interventions designed to improve compliance with evidence-based guidelines for preventive care could have an important influence on the health of Canadians. In addition, interventions designed to reduce the ordering of inappropriate tests in delivering preventive care have the potential to offset some of the intervention costs.

### Economic evaluations of outreach facilitation

By its nature, outreach facilitation is multifaceted. In the United Kingdom and the United States specially trained nurse facilitators organized preventive care in busy practitioners' offices through outreach visits and using approaches such as academic detailing, chart audit and feedback for the prevention and early detection of cardiovascular disease and cancer [22,23]. Very few evaluations of outreach facilitation have studied the costs of delivering these interventions. The Cochrane Effective Practice and Organization of Care Group has concluded that outreach visits are effective, however, cost-effectiveness needs to be determined [24]. Soumerai and Avorn have suggested that the savings from outreach facilitation may outweigh the costs if the intervention is targeted at inappropriate and costly practice behaviour [25,26].

Cockburn and colleagues conducted a randomized controlled trial in which the effectiveness of three approaches to marketing a quit smoking intervention kit to physicians was evaluated [27]. They conclude that educational outreach facilitators do not appear to be cost effective strategies for distributing smoking interventions. The actual use of the kit by the physicians for their smoking patients did not differ significantly across groups. However, there was a trend toward higher use in the facilitation group for one of the components of the kit as compared to those who received the kit by courier or standard mail.

Conversely, McCowan et. al. conducted a randomized controlled trial to examine the effect of a facilitator intervention on the management of children with asthma by family physicians [28]. They found that the facilitator intervention reduced asthma care costs in the intervention group as compared to the control resulting in an overall net saving of 12,000 (U.K. 1991) pounds or one pound less per child per annum. The facilitator accomplished this by inserting guidelines for the management of asthma into intervention practices' case records. The authors estimate that the net savings to the health system would recoup the facilitator's salary at 1991 rates.

The literature on the costs of outreach facilitation is limited. It can be argued that facilitation is a costly intervention [27]. However, a costly intervention that achieves success may be preferred to a cheaper one that demonstrates very little or has no lasting effect. More research on the costs of successful facilitation and other effective alternative interventions to outreach facilitation is necessary.

### Intervention description

The nurse facilitation in our study was a tailored multifaceted approach to getting evidence into action. Three nurse facilitators focused on the educational, attitudinal and organizational barriers to change in the practice setting and tailored a multi-component intervention to the specific needs of the practice [22,23,29,17-34]. The facilitators completed a 30-week intensive training program before being assigned to intervention practices. The training covered an orientation session, medical office computer systems, medical practice management, prevention in primary care, evidence-based medicine, and facilitation and audit skills development. Approximately 28 hours per week were spent in training and 7 hours per week in preparation and planning. Six of the 30 weeks of training were spent applying skills in a primary care office setting.

The intervention period was 18 months ending December 1998. During this period each intervention practice was visited an average of 33 times (range 21 to 50) at an average visit length of one hour 45 minutes. The facilitators delivered primarily three intervention strategies to improve preventive care: chart audit and feedback, educational consensus building, and reminder systems. They discussed the strategies with the physicians and practice staff, working with them to adapt the strategies to the practice needs and wishes. All 22 of the intervention practices participated in an initial audit and received feedback on preventive care practice patterns. Twenty practices requested subsequent audits and analyses of data to follow their rates of performance. All of the practices were involved in meetings with the facilitator to identify opportunities for improvement, assess needs, receive and discuss critically appraised evidence from the literature for the preventive maneuvers, and select strategies for improving preventive care performance. All of the intervention practices implemented some form of a reminder system as a strategy to improve performance. Eighteen practice sites implemented a preventive care flow sheet; two sites used a chart stamp; and two sites implemented a computerized reminder system.

The facilitators provided management support to practices and followed a quality improvement framework similar to that proposed by Leininger and colleagues [30]. For each practice the facilitators were to: (1) present preventive performance rates prior to the intervention, (2) facilitate the development of a practice policy for preventive care, (3) assist in the setting of goals and desirable levels of performance, (4) assist in the development of a written plan for implementing preventive care, (5) assist in the development and adaptation of tools and strategies to implement the prevention plan, (6) facilitate meetings to assess progress and modify the plan if necessary, and (7) conduct performance feedback to measure the effect of changes made.

The facilitators had no interaction with control practices. The latter were told that they were involved in a study on prevention but were not told which preventive manoeuvres were being measured. More details on the multi-component nature of the intervention are published elsewhere [35].

### Setting

The Prevention Facilitator intervention involved Health Service Organizations (HSOs) in Ontario. HSOs are community primary care practices that have a payment system based primarily on capitation and not fee-for-service. At the time of the study, there were 72 physician-sponsored HSOs located at 100 different sites in Ontario. The study involved 106 physicians from 45 HSOs. All physicians gave informed consent to participate in the trial.

### Intervention outcomes

The goal of the intervention was to increase those preventive manoeuvres supported by evidence as appropriate and decrease those preventive manoeuvres supported by evidence as inappropriate. Eight grade A and B, and 5 grade D preventive manoeuvres were chosen by a panel of practicing family physicians from the Canadian Guide to Clinical Preventive Health Care[3] to represent a broad spectrum for both male and female adult patients (see Table 1). The grade A and B manoeuvres are supported by evidence as appropriate and the grade D manoeuvres are supported by evidence as inappropriate. As determined by chart audit, an absolute change overtime of 11.51% in preventive care performance in favour of intervention practices was achieved. More detailed outcome results of the randomized controlled trial are reported elsewhere [36].

**Table 1:** Preventive manoeuvres studied

**Level of Evidence**	**Preventive Manoeuvre**
**A^Ω ^& B^¥^-categories**: (Appropriate)	1. Folic acid for primary prevention of neural tube defects
	2. Smoking cessation and nicotine replacement
	3. Treatment for Hypertension
	4. Mammography and exam in women over 50
	5. STD screening for high risk groups
	6. Papanicolaou smears for sexually active women
	7. Influenza vaccination to patients 65 and older
	8. Blood pressure measurement for patients 21 to 64 years of age
	
**D^ψ^- category: **(Inappropriate)	1. Proteinuria screening for general population
	2. Blood glucose for the general population
	3. Prostate-specific antigen testing for men over 50
	4. Chest radiography
	5. Mammography in women under 50

^Ω ^There is excellent evidence from repeated randomized controlled trials to support the manoeuvre.

^¥ ^There is good evidence from cohort and case-control studies to support the manoeuvre.

^ψ ^Not recommended on the basis of fair evidence not to perform the manoeuvre.

**Table 2:** Demographic profile comparison of intervention and control practices

**Measure**	**Intervention Group (N = 22)**	**Control Group (N = 23)**	**Significance (P value)**
Percentage of Group Practices	77.3%	60.9%	.34
Percentage Teaching Affiliated	54.5%	52.2%	1.00
Percentage in communities greater than 50,000	86.4%	65.2%	.17
Mean number of physicians in group practices	2.91	2.70	.71
Mean number of registered nurses in practices	1.16	1.64	.48
Mean year of graduation from medical school	1975	1975	.92
Mean proportion of female physicians	12.6	20.4	.37
Mean roster size	4317	3874	.55
Mean number of patients seen per day	34.4	33.0	.59
Percentage of female patients served	53.4	53.8	.89
Mean age of patients served	46.4	46.8	.87

**Table 3:** Comparison of intervention and control practices on delivery of preventive manoeuvres to eligible patients post intervention (N = 4501)

**Preventive Manoeuvres**	**Proportion of Eligible Patients**		
	Intervention (n = 2201)	Control (n = 2300)	Significance
**A & B Manoeuvres**	% (N)	% (N)	
Folic Acid Pre-conception	15.4% (325)	4.9% (369)	.0001
Cessation Counselling	41.7% (571)	40.6% (549)	N.S.
Mammography 50 to 69	68.3% (325)	57.5% (358)	.005
Hypertension Treatment	79.7% (169)	82.7% (185)	N.S.
STD Screening	23.3% (382)	19.1% (366)	N.S.
BP Measurement	74.6% (1666)	72.5% (1781)	N.S.
FLU Vaccination	66.0% (692)	53.8% (652)	.0001
Cervical Cytology	66.2% (826)	60.2% (958)	.01
			
**D Manoeuvres**			
Blood glucose screening	32.8% (1844)	38.7% (1980)	.0001
PSA Testing	30.6% (379)	30.0% (393)	N.S.
Mammography 40 to 49 ^1^	11.6% (267)	9.1% (309)	N.S.
Chest X-Ray	3.7% (571)	4.9% (549)	N.S.
Urine proteinuria screening	16.5% (1772)	29.8% (1887)	.0001

^1^This was a grade D manoeuvre at the time of the study; the Canadian Task Force on Preventive Health Care has recently changed it to a Grade C manoeuvre.

We wanted to estimate the cost savings associated with an effective outreach facilitation intervention designed to reduce inappropriate and improve the delivery of appropriate preventive care.

## Methods

The analysis was part of a randomized controlled trial to determine if outreach facilitation was effective in improving prevention in primary care. The design of the trial and details of the method have been described in full elsewhere [36]. Practices were randomized to either intervention (22 practices) or control status (23 practices).

### Patient eligibility

Patients eligible for the intervention were greater than 19 years of age, asymptomatic and had received the intervention for screening and prevention purposes.

### Cost analysis methodology

We performed a cost-consequences analysis to evaluate the outreach facilitation intervention [37]. We determined the incremental costs of the preventive manoeuvres performed and the overall cost of the intervention. We estimated the cost-savings as a result of having improved appropriate preventive care and reduced inappropriate preventive care between intervention and control conditions. All of the appropriate and inappropriate preventive manoeuvres from the trial were included in the analysis, not only those that showed significant improvement. Multiple data sources were used to determine the costs of preventive procedures, treatment, and efficacy of preventive manoeuvres (see Tables 4 and 5). We determined the costs and savings associated with outreach facilitation by calculating the mean difference between intervention and control groups in the number of eligible patients screened or treated for appropriate preventive manoeuvres and the mean difference in the number of eligible patients not screened for inappropriate preventive care. The cost-consequences analysis is conducted from the perspective of the Ontario Government. All costs are presented on the basis of one year rather than over the 18-month period of the intervention to correspond with the government one-year planning and budgeting cycle. Costs were converted into Canadian dollars using the nominal rate method and adjusted for inflation (1999 current dollars) where necessary. The net cost savings per patient and per facilitator are presented.

**Table 4:** Input variables and cost estimates for appropriate manoeuvres (1999 dollars)

**Variable**	**Value and Range^a^**	**Source**
**A & B Manœuvres**		
Folic Acid		
Difference in no. of eligible patients counseled	1495 (854, 2136)	(36)
Probability of reducing neural tube defects	.00058	(55)
Life-time treatment costs of Spina Bifida	$201,822^b^	(51)
		
Smoking Cessation Counselling/NRT		
Difference in no. of eligible patients counseled	253 (-1072, 1577)	(36)
Efficacy of NRT	.06	(3)
Incidence of lung cancer in smokers	.0024	(48)
Treatment costs for lung cancer	$7,074	(56)
		
Mammography 50 to 69 years of age		
Difference in no. of eligible women screened	1513 (505, 2521)	(36)
Incidence of breast cancer	.003165	(42)
Cost of a mammogram	$76.54	(57)
Treatment costs saved for each breast cancer	$2,522	(42)
		
Hypertension Treatment		
Difference in no. of eligible patients treated	-145 (-739, 449)	(36)
Efficacy of treatment of stroke	.42	(3)
Incidence of stroke	.0018	(47)
Efficacy of treatment of heart disease	.14	(3)
Incidence of heart disease	.00196	(47)
Treatment cost per case for stroke	$3,815	(56)
Treatment cost per case for heart disease	$3,303	(56)
		
STD Screening		
Difference in no. of eligible patients screened	645 (-253, 1542)	(36)
Cost of gonorrhoeae and chlamydia culture	$55	(57)
Incidence of gonorrhoeae or Chlamydia infection	.08	(49)
Pelvic inflammatory disease (PID) prevented	.14	(50)
Treatment cost per case for PID	$1,782	(49)
		
Flu Vaccination		
Difference in no. of eligible patients vaccinated	3364(1928,4799)	(36)
Cost of flu vaccination	$3.75	(43)
Pneumonia hospitalizations averted	4.1 per 1000	(43)
Chronic respiratory hospitalizations averted	10.4 per 1000	(43)
Congestive heart failure hospitalizations averted	2 per 1000	(43)
Emergency room visits avoided	21.6 per 1000	(43)
Emergency room visit cost	$76.00	(44)
Cost of pneumonia hospitalization	$4,462	(56)
Cost of chronic respiratory hospitalization	$4,445	(56)
Cost of heart failure hospitalization	$5,417	(56)
		
Cervical Cytology		
Difference in no. of eligible patients screened	2196 (559, 3833)	(36)
Cost of PAP test	$57.17	(57)
Incidence of cervical cancer	.00013	(46)
Treatment costs saved for each women screened	$9,813	(41)

^a ^95% Confidence Intervals

^b ^10% discount

**Table 5:** Input variables and cost estimates for inappropriate manoeuvres (1999 dollars)

**Variable**	**Value and Range^a^**	**Source**
**D Manoeuvres**		
Blood Glucose Screening		
Difference in eligible patients not screened	4709 (2329,7089)	(36)
Specificity of blood glucose test	89%	(3)
Cost of fasting glucose test	$10.34	(57)
Cost of glucose tolerance test	$23.26	(57)
Cost of HgA1c	$19.12	(57)
		
PSA Testing		
Difference in eligible patients not screened	95 (-932, 1122)	(36)
Cost of PSA Test	$25	(57)
Specificity of PSA test	40%	(3)
Cost of Biopsy*	$232.83	(58)
		
Mammography 40 to 49		
Difference in eligible patients not screened	295 (-296, 887)	(36)
Cost of mammogram	$76.54	(57)
Incidence of breast cancer	.001616	(42)
Specificity of mammogram	96.5%	(3)
Cost of Biopsy	$2,164	(42)
		
Chest X-Ray		
Difference in eligible patients not screened	276 (-271, 822)	(36)
Cost of chest x-ray	$233	(40)
		
Urine Proteinuria Screening		
Difference in eligible patients not screened	9947 (7934,11961)	(36)
Specificity of urine dipstick test	95%	(3)
Cost of Urine culture and urinalysis	$27.18	(57)

^a ^95% Confidence Intervals

*Includes intracavitary ultrasonography, ultrasound guidance of biopsy and needle biopsy of the prostate

### Intervention costing methodology

The actual cost of the intervention over 18 months was gathered from administrative expenditure records for labour, supplies, telephone and travel. The investigation team determined that the cost of training the nurse facilitators was important to include but that the investment in training was worth more than the 18-month period of the intervention. Therefore, the cost of the training of the three nurse facilitators was depreciated over a 5-year life at a discount rate of 5% using the double-declining balance method for the 18-month period of the intervention. To correspond with the government planning and budgeting perspective, research costs were not included but supervision was. The justification for excluding research costs and including supervision was to model the program as if it were implemented as an actual government sponsored intervention. As a government sponsored program there would be no research salary and operations costs, but there would still be the need to supervise the facilitators. In addition, the cost of physician time for administering preventive manoeuvres was not included. The rationale for excluding physicians' time for administering preventive manoeuvres is based upon the fact that the physicians were not reimbursed on a fee-for-service basis but rather were reimbursed on a capitation basis and the intervention did not result in an increased number of visits keeping costs the same in both the intervention and control groups. Within a capitation based system, physicians are reimbursed according to the size of their patient roster and not by fee-for-service. Physicians within a capitation based system do not receive additional fees for providing preventive services, nor do they receive any additional remuneration from seeing a patient more frequently. Therefore, this is not an additional cost to the Ontario provincial government.

### Preventive manoeuvre costs

Using the most recent fee schedule for laboratory services, published by the Ontario Ministry of Health in April of 1999, we calculated the direct costs of performing seven appropriate and five inappropriate measures. Where cost estimates were unavailable from these two sources, published estimates for the cost of bilateral mammography[38], administering a flu shot[39], a chest x-ray[40] and cervical cancer screening[41]were used. The cost of folic acid, nicotine replacement therapy, hypertensive medication and antibiotics were not included since from the perspective of the Ontario government we determined that these costs would be substantially paid for directly by patients. The measurement of blood pressure was included as a case finding manoeuvre for hypertension as described by the Canadian Task Force on Preventive Health Care [3] but was not costed as 80% of eligible patients in treatment and control groups received a blood pressure measurement and as a consequence did not impact the cost analysis. Calculations were performed using Microsoft Excel Version 7.0.

Direct costs associated with inappropriate tests avoided are considered savings to the government whereas the costs associated with increasing the number of appropriate tests are considered costs to the government (see Tables 4 and 5). The direct cost for each inappropriate test, according to the Ontario Ministry of Health, includes not only the test cost for performing the manoeuvre but also a "Patient Documentation and Specimen Collection Fee" which can be applied to a test or series of tests performed for one patient. The costs for appropriate tests were gathered from the estimates of other relevant cost studies (see Table 4). For the purpose of calculating costs, the total population of 184,670 patients as of November 1998 for all 45 HSO practices was used to estimate the total number of intervention patients eligible (90,283) to receive a test according to the patients eligible for the selected tests from the study sample of 4,501 patients (2,201 intervention patients, 2300 control patients).

The cost of the initial visit to the physician was not included given that this cost would have been included whether a test was performed or not and these costs would be similar between the intervention and control groups and thus would not significantly impact the cost analysis. In addition, direct costs to the patient such as travel time to a laboratory to have a blood test have not been included. However, down-stream costs (follow-up visits and further tests) for the subsequent follow-up of false positive results for all screening manoeuvres where appropriate have been included. The down-stream costs included the cost of biopsies to determine breast cancer and prostate cancer as well as the cost of follow-up glucose tolerance tests to screen for diabetes. Any down-stream costs associated with other preventive manoeuvres were not considered for the analysis.

The number of expected false positive screening tests was determined from the specificity of a blood glucose test (89%), a urine dipstick test for protienuria (95%), and a PSA test (40%) [3]. A panel of five family physicians with approximately 100 years of combined practice experience determined through consensus the proportion of follow-up tests and visits needed. For estimating the total costs of performing follow-up on initially positive results for blood glucose, it was agreed that 40% would receive only a glucose tolerance test and 60% would receive both a fasting blood glucose and an HgA1c. For estimating the total costs of performing follow-up on positive urine protein tests, it was assumed that 100% of patients would receive both a urine culture and a microscopic urinalysis and a 24-hour urine. The documentation and specimen collection fee of $7.75 was only included once for each patient visit. For estimating the total costs of performing follow-up on a positive PSA result, it was assumed that 90% of patients would be referred to an urologist [58]. The cost of family physician follow-up visits was not included given that the HSO physicians were not reimbursed on a fee-for-service basis. The input variables and cost estimates for appropriate and inappropriate manoeuvres are provided in Tables 4 and 5.

### Preventive manoeuvre savings

The input variables and cost estimates for savings associated with preventive manoeuvres are provided in Tables 4 and 5. The difference in the number of patients receiving preventive manoeuvres between the intervention and control practices gave us the increase in savings to the government. Mammography can reduce breast cancer mortality by 20% to 30% [3]. For savings associated with Mammography in women 50 to 69, Salzmann et al. [42] was used to determine the difference in lifetime treatment costs of screened and unscreened women with breast cancer. The estimated difference is $1,682 $US 1995 or converted and inflated to 1999 dollars $2,522 $Can less for the screened group. It is assumed that the incidence of breast cancer in women 50 to 69 is 3.165 per thousand [42].

For savings associated with administering flu vaccine in the elderly, Nichol et. al.[43] provided the data on the number of hospitalizations of the elderly averted due to flu vaccination for pneumonia (4.1 per 1,000), chronic respiratory conditions (10.4 per 1,000), congestive heart failure (2 per 1,000) and the number of emergency room visits avoided (21.6 per 1,000). The estimated cost of an emergency room visit of $76.00 $Can 1999 as determined by Jacobs and Hall was used [44]. The Ontario Case Costing project provided the average length of stay and the total cost of hospitalization for pneumonia ($4,462 $Can 1999), chronic respiratory conditions ($4,445 $Can 1999) and congestive heart failure ($5,417 $Can 1999) for patients 65 years of age and older.

Performing Pap tests every three years reduces invasive cervical cancer by 91.2% [45]. Fahs et al. [46] provided the cervical cancer incidence rate (10 per 100,000 women) and Helms et al.[41] provided the difference in cervical cancer treatment at stage of diagnosis. The difference between the net costs of treatment for patients in the initial stages of cervical cancer and more invasive cancer is $6,368 ($US 1988) or $9,813 ($Can 1999).

For determining savings associated with smoking cessation counselling and treatment of hypertension, the Canadian Task Force on Preventive Health Care [3] provided the percent efficacy of cessation counselling and the treatment of hypertension for stroke and heart disease. The Canadian Institute for Health Information [47] provided the incidence of stroke and heart disease and Statistics Canada provided the incidence of lung cancer in smokers [48]. The Ontario Case Costing project provided the total cost for lung cancer hospitalization ($7,074 $Can 1999), stroke hospitalization ($3,815 $Can 1999), and hospitalization for heart disease ($3,303 $Can 1999) for determining overall treatment cost savings.

For savings associated with STD screening, Gift et. al.[49] provided the figure for the incidence of gonorrhoeae or chlamydia infection for eligible women as well as the cost to treat a case of pelvic inflammatory disease (PID) and the Centers for Disease Control and Prevention provided the figure for the prevention of pelvic inflammatory disease from treatment [50].

Finally, for determining the costs averted as a result of increasing folic acid intake, Waitzman et. al.[51] provided the data on the net direct medical costs associated with Spina Bifida ($123,485 $US 1996 or $201,822 $Can 1999) and the Canadian Task Force on Preventive Health Care and the CDC provided the estimates for the reduction in neural tube defects due to folate, an estimated 50% of all neural tube defects or 500 per million births [3].

After calculating the current (1999) cost of the intervention and the cost of the increase in delivery of recommended preventive manoeuvres we calculated the potential direct savings to the government for increased appropriate and decreased inappropriate preventive care by multiplying the costs with the estimated difference in patients screened or treated using the estimates and input variables provided in Tables 4 and 5. We also calculated a low and high estimate of costs using the 95% confidence intervals of the difference in patients screened between intervention and control groups. These calculations are summarised mathematically in Appendix – [see Additional file 1].

### Sensitivity analysis

To account for uncertainty in the cost-consequences analysis, a 1,000 iteration Monto Carlo simulation was conducted to determine the impact of the range of input cost parameters on the net savings to government using Microsoft Excel. The cost input parameters included the normally distributed range of values between the 95% confidence intervals for percent difference between intervention and control practices on the preventive manoeuvre performance outcomes. These parameters are considered the most important in influencing overall costs and cost savings in the analysis. The ranges of randomly chosen estimates of each parameter were varied simultaneously to determine the impact on costs and cost savings. Finally, the ranges of input parameters were assessed individually on the ranges of the net savings outcome generated by the simulation to generate a Tornado diagram. Percent variance was used to determine the degree to which the outcome net savings was sensitive to the values of each input parameter and to determine which input parameter had the greatest impact on net savings.

## Results

Intervention (n = 22) and control (n = 23) group practices did not differ significantly on any of the demographic characteristics presented in Table 2.

### Intervention effect

Table 3 presents the proportion of eligible patients that received recommended (A & B Manoeuvres) and inappropriate (D Manoeuvres) preventive care for both intervention and control groups of patients after the outreach facilitator intervention.

For inappropriate testing there was a statistically significant but small reduction in the proportion of eligible patients that received a random blood glucose test to screen for diabetes (32.8% vs. 38.7%) and a larger reduction for urine protein testing to screen for kidney disease (16.5% vs. 29.8%). There were no significant differences for PSA testing to screen for prostate cancer, mammography for women 40 to 49, or chest radiography to screen for lung cancer in smokers. For appropriate preventive manoeuvres there was a statistically significant difference for folic-acid counselling, mammography for women 50 to 59, flu vaccination, and cervical cytology.

### Intervention costs

Table 6 provides data on the costs of the outreach facilitator intervention in 1999 dollars. The 18-month intervention cost a total of $357,583.00 for three nurse facilitators including all travel, telephone, supplies and supervision or $238,388 per year. Telephone costs include the cost of a cellular phone as well as the long distance costs of a home telephone. Supply costs include the costs of home-office supplies as well as the cost of materials for intervention purposes in the practices. The cost of the intervention on a yearly basis equates to $10,835 per intervention practice (n = 22) or $4,584 per intervention physician (n = 52) or $328 per visit based on an average of 33 visits to the practice per year.

**Table 6:** Intervention costs over 12 months

**Cost Item**	**1999 Dollars**
Staff training	$20,450
Salaries & Benefits	$178,200
Supplies	$7,060
Telephone	$8,629
Car Mileage & Insurance	$12,049
Supervision	$12,000
	
INTERVENTION COST	$238,388

Table 7 provides the costs of providing recommended preventive care. The intervention resulted in 1,513 more mammograms having been done which is estimated to be an additional cost to the government of $77,192 per year with a range of $25,743 to $128,641 depending on the number of mammograms. 645 additional cultures for gonorrhoeae and chlymadia were carried out in the intervention arm resulting in an estimated $23,631 in costs. In addition, 3,364 more flue shots were provided in the intervention arm of the trial costing $8,409 per year and 2,196 more women received a PAP test at a cost of $83,679 per year (see Table 7).

**Table 7:** Costs associated with the increase of appropriate preventive manoeuvres (1999 Dollars)

**Manoeuvre**	**Baseline**	**Low**	**High**
STD Screening	$23,630.87	-$9,268.28	$56,530.01
Mammography 50 to 59	$77,192.11	$25,742.87	$128,641.34
Influenza shot	$8,409.23	$4,820.83	$11,997.62
Pap Test	$83,679.41	$21,286.01	$146,072.81
			
TOTAL	$192,911.62	$42,581.43	$343,241.78

The total cost to the government for the intervention and additional manoeuvres performed was $431,300 (95% CI: $280,969 – $581,630) on a yearly basis, that equates to $19,604 per intervention practice, or $143,766 per facilitator or $4.67 per HSO patient rostered (see Table 10).

### Cost savings

Table 8 gives the estimated cost savings in 1999 dollars as a result of having significantly reduced inappropriate care in the intervention group for unrecommended screening manoeuvres. For the laboratory and diagnostic services cost model extrapolated to the entire eligible patient roster for intervention practices there is an estimated $148,568 (95% CI: -$175,898 – $473,035) in total savings including follow-up tests. This represents 34% of the costs of the outreach facilitator intervention and reduces the program to a cost of $282,732.

**Table 8:** Savings associated with the reduction of inappropriate preventive care (1999 Dollars)

**Manoeuvre**	**Baseline**	**Low**	**High**
Chest X-Ray	$42,827.36	($42,065.12)	$127,719.83
Mammography 40 to 49	$16,107.12	($16,131.58)	$48,345.82
PSA Testing	$16,125.11	($158,156.15)	$190,406.37
Blood glucose	$59,990.54	$29,672.59	$90,308.49
Urine Protein	$13,518.31	$10,782.33	$16,254.29
			
TOTAL	$148,568.44	($175,897.93)	$473,034.80

Table 9 provides the cost savings to the government as a result of averted hospitalizations and treatment costs. There is an estimated $455,464 (95% CI: $237,935 -$672,992) per year in averted hospitalization and treatment costs as a result of increasing the provision of recommended care to eligible patients. Most of the cost averted ($254,633 per year) is due to the estimated reduction in hospitalizations for pneumonia [14], acute chronic respiratory conditions [35], and cases of congestive heart failure [7]. The treatment costs saved of an estimate of one case of Spina Bifida equated to $174,999. Costs averted due to hospitalizations and treatment equate to $20,703 per practice, $151,821 per facilitator, and $4.93 per patient.

**Table 9:** Savings from the provision of appropriate preventive care (1999 Dollars)

**Condition**	**Baseline**	**Low**	**High**
Breast Cancer	$12,075.20	$4,026.97	$20,123.44
Influenza	$254,633.54	$145,976.01	$363,291.07
Neural Tube Defects	$174,999.39	$99,997.21	$250,001.56
Cervical cancer	$2,154.49	$548.05	$3,760.92
Lung Cancer	$122.27	($518.51)	$763.06
Heart Disease	($808.13)	($4,116.12)	$2,499.86
STD Treatment	$12,862.75	($5,044.91)	$30,770.42
Stroke	($575.90)	($2,933.28)	$1,781.48
			
TOTAL	$455,463.61	$237,935.42	$672,991.81

**Table 10:** Estimated cost savings

	**Baseline**	**Low**	**High**
Costs			
Intervention Cost	$238,388	$238,388	$238,388
Manoeuvre Costs	$192,911.62	$42,581.43	$343,241.78
Total Costs	$431,299.62	$280,969.43	$581,629.78
			
Savings			
Inappropriate manoeuvre savings	$148,568.44	($175,897.93)	$473,034.80
Treatment cost savings	$455,463.61	$237,935.42	$672,991.81
Total Savings	$604,032.05	$62,037.49	$1,146,026.61
			
NET Savings	$172,732.44	($218,931.95)	$564,396.83

Table 10 shows that the cost of the program over one year combined with the increased costs associated with delivering more recommended preventive care is $431,300. The costs averted from reducing inappropriate tests and the reduction in hospitalization and treatment are $604,032. This is a net cost savings to the government of $172,732 (95% CI: -$218,932 – $564,397) per year as a result of the intervention with 22 practices and equates to $1.87 per patient saved or $3,321 per physician, an estimated return on the intervention and investment in additional manoeuvres of 40%. Based on a Consumer Price Index of $1.11 the net cost savings to the government in 2003 dollars are $191,733 per year or $8,715 per practice, $3,687 per physician, or $2.08 per patient.

### Sensitivity analysis

**Figure 1:**
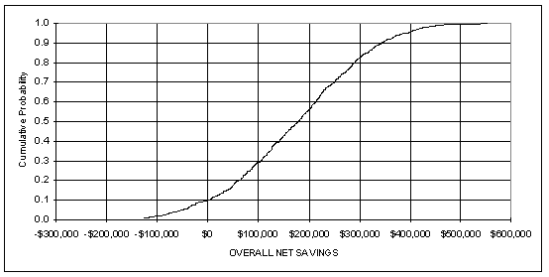
Distribution of outcome for net savings

**Figure 2:**
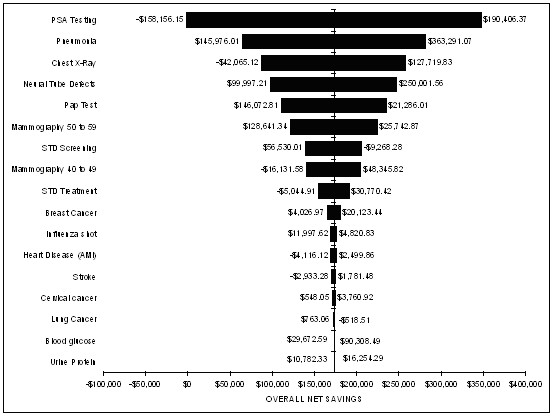
Sensitivity analysis for net savings

The sensitivity analysis using Monte Carlo simulation revealed that the mean net savings in 1999 dollars to the government would be $175,510 and that 90% of the expected net savings to the government would fall between $-43,000 and $394,000 (see Figure 1). Based upon 1,000 iterations of the cost model and the assumptions regarding input costs, it is unlikely that the government would not receive some return on its investment in outreach facilitation. Finally, Figure 2 shows the relationship between each of the input variables to net savings. As would be expected net savings is very sensitive to PSA testing with the more men tested the less net savings accounting for 47.4% of the variance. Flu vaccination also impacts net savings in terms of pneumonia prevented and accounting for 18.4% of the variance in net savings. Due to the poor specificity of the PSA test and the non significant effect of the intervention to reduce PSA tests in eligible men, the cost associated with the PSA test varies considerably and its reduction is critical for attaining cost savings whereas the increase of PSA tests is negatively associated with savings. In contrast, PAP testing and mammography for 50 to 59 year old women in the cost model are not as negatively associated with net savings despite the increase in costs as more of these manoeuvres are performed.

## Discussion

The primary objective of this study was to determine if decreasing inappropriate preventive tests and increasing appropriate testing performance could offset the cost of an outreach facilitation intervention to improve preventive practice. The significant reduction in inappropriate testing and increase in appropriate testing resulted in net savings of $191,733 per year in 2003 dollars to the government or a return on investment of 40%. The limitations of the cost analysis are typical to this type of economic evaluation [37]. They include:

• the perspective of the government necessitated not having included all possible costs in the model. For example, the cost for patient time, travel or patient discomfort and anxiety associated with the manoeuvre were not included;

• the total indirect costs associated with hospitalizations averted were not included;

• the estimate of the frequency of downstream events was based on a panel of experts;

• the estimate for screening for cervical cancer was based on a yearly screening rather than once every three years;

• downstream costs were estimated and included in the model for follow-up visits as a result of a false positive test only. Other possible downstream costs such as visits to other allied health professionals or consults to specialists were not included;

• the benefit of inappropriate screening tests for some patients and the associated cost savings have been ignored; and

• rates of delivery for preventive screening tests were from a randomized controlled trial in a HSO setting. Therefore, caution must be used when generalizing the potential cost savings to other settings.

Our cost estimates in this analysis are conservative since patient costs and other downstream costs were not included. Nonetheless, the analysis shows that all of the costs of the outreach facilitator intervention can be recouped as a result of having reduced inappropriate testing and increased appropriate testing for the manoeuvres under study. Similarly, McCowan et. al. were able to show that a facilitation intervention was able to improve primary care asthma management and that the cost savings to the health care system could completely offset the annual salary of one facilitator serving a large number of family physicians [28]. McCowan's study also included the costs associated with hospital admissions and secondary costs and involved the improvement in treatment of an acute illness and not prevention in primary care. Including the downstream costs associated with inappropriate tests averted for the outreach facilitator intervention has allowed for an additional 35% in estimated cost savings and the inclusion of costs averted associated with appropriate testing has completely offset the cost of outreach facilitation.

The successful outreach facilitator intervention described in this study was a very intensive intervention with each practice being visited an average of 33 times over 18 months. This compares to other successful trials such as Dietrich et. al. [23] where outreach facilitators visited only 3 times over a three-month period at an average of 120 minutes per visit and Hulscher et. al. [32] where facilitators visited practices an average of 25 times with an average duration of only 73 minutes. Both of these studies used outreach facilitation to improve preventive practice for a number of different maneuvres. Unfortunately, the cost of outreach facilitation was not included in these less intensive studies.

The cost of an outreach facilitator in our study per year was over $4,497 per physician but resulted in an overall net savings to the government of $3,289 per physician. In comparison, Cockburn et. al. tested an educational outreach facilitator intervention to improve physician smoking cessation counselling performance which cost $A142 in 1992 per practitioner [27]. However, the facilitator only visited each physician twice at an average of 12 minutes per visit or $A72 per visit and achieved very little in the way of improved outcomes. As a consequence, unsuccessful outreach facilitation was shown to be not cost-effective compared to other alternatives. In our intervention the cost per visit was $590 and nothing after having adjusted for cost savings associated with a successful intervention. Our intervention was targeted at changing the entire practice and not just physician behaviour for a number of preventive measures, and as a result more time was spent on-site and more visits were required. More research is necessary to determine the most appropriate intensity of intervention for a given level of outcome.

The Cochrane Effective Practice and Organization of Care Group has compiled evidence that supports outreach visits combined with additional interventions as effective in improving professional practice and health outcomes [13]. Our study has demonstrated the effectiveness of outreach facilitation in improving overall preventive care performance. This is the first cost-consequences analysis of an outreach facilitation intervention that we are aware of and we have shown that the savings attributable to the reduction in inappropriate testing and increases in appropriate testing can offset all of the intervention cost and in fact result in a net savings to the government of approximately two dollars for every rostered patient. Further, the sensitivity analysis has revealed that the likelihood of net savings to the government is substantial despite variation in the inputs. However, it has also revealed that the appropriate primary preventive manoeuvres such as vaccination of the elderly contribute more to net savings to the government than some secondary preventive manoeuvres such as Pap testing and mammography. The net savings associated with primary prevention help to cover the net costs to the government associated with secondary prevention. As well, in the case of PSA testing even greater cost savings may be possible by significantly reducing this and other inappropriate tests and the consequent downstream costs.

Filak et. al. [52] calculated the lifetime charges of office visits, procedures, laboratory tests, and patient purchases required to comply with the US Preventive Services Task Force screening recommendations. They determined that the lifetime charges in 1999 US Dollars for all required preventive services ranged from $5,432.60 to $7,529.60 for men and from $15,307.10 to $18,525.10 for women. If physicians could deliver all the necessary preventive care to their patients, the costs over a lifetime are very reasonable in comparison to all of the costs associated with treating a stage I ($14,000) or stage IV ($64,000) cancer of the breast in a woman over 50 [53] or a case of pneumonia ($35,700) caused by influenza in a person 65 years old [54]. The outreach facilitator intervention was effective in improving the uptake of preventive manoeuvres shown to be very cost-effective with the added benefit of reducing preventive manoeuvres that are less cost-effective. If the estimated 9,850 family physicians in Ontario received the benefit of outreach facilitation, the estimated savings to the government equate to $36.3 million (2003 dollars). However, this study involved HSO physicians who may not be representative of all family physicians and research has demonstrated that the intervention does not work in chaotic practices [59].

## Conclusion

This paper has provided information on the cost of outreach facilitation and the potential for cost savings to the health system. The results can be considered an underestimate of the true potential cost savings given that not all the costs associated with inappropriate and appropriate preventive care were considered. Further, this was an efficacy trial and as a consequence there is potential for reducing the cost of the intervention through efficiency improvements such as increasing the number of practices per facilitator as well as savings in administration and training through economies of scale. In addition, additional savings to the health system may be possible through on-going outreach facilitation for chronic illness care.

There are no magic bullets to changing primary care practice patterns [9]. However, a facilitation approach that incorporates a number of intervention strategies tailored to the environment and needs of the practice holds promise. The literature has shown that outreach facilitation is one of the most effective means of improving the delivery of primary care preventive health services [13]. Further economic evaluations of outreach facilitation and other intervention alternatives are needed to assist in important public policy and administrative decision-making on getting preventive care guidelines into practice.

## Competing interests

The research project was funded by a grant from the Ontario Ministry of Health. All of the authors were involved in the research project to evaluate the effectiveness of outreach facilitation in improving the delivery of preventive health care guidelines. WH and JL are practicing family physicians, professors with the University of Ottawa and were investigators on the project. NB is a PhD candidate at the University of Waterloo and was employed as the project co-ordinator for the evaluation.

## Authors' contributions

WH is the Principal Investigator for the Closing the Loop on Prevention project. WH and JL conceived the economic evaluation study, participated in the study design, preparation of first drafts and critical revisions of the manuscript and contributed to all other aspects of the study. NB contributed to the economic study design, acquired the economic data, performed the economic analysis and interpretation, prepared, and revised the manuscript.

## Pre-publication history

The pre-publication history for this paper can be accessed here:



## Supplementary Material

Additional File 1 Mathematical Summary Click here for file
