# Supplementary material for: Cost savings associated with improving appropriate and reducing inappropriate preventive care: cost-consequences analysis
Source: BMC Health Serv Res. 2005 Mar 9;5:20. doi: 10.1186/1472-6963-5-20 (PMC1079830; doi:10.1186/1472-6963-5-20)
Supplement: Additional File 1 — Mathematical Summary [file 1472-6963-5-20-S1.doc]

# Additional files

### Additional file 1 – Mathematical Summary

## Costs Associated with the Increase of Appropriate Preventive Manoeuvres

**1) STD Screening = ((STDdiff x STDcost)/18) x 12[[1]](#footnote-2)**

STDdiff = patient population eligible x the difference in patients screened between intervention and control groups

STDcost = current cost of gonorrhoeae and Chlamydia culture

**2) Mammography 50 to 59 = ((MAMMdiff x MAMMcost)/18) x 12**

MAMMdiff = patient population eligible x the difference in patients screened between intervention and control groups

MAMMcost = current cost of a bilateral mammogram

**3) Influenza Vaccination = ((FLUdiff x FLUcost)/18) x 12**

FLUdiff = patient population eligible x the difference in patients vaccinated between intervention and control groups

FLUcost = current cost of a influenza shot

**4) PAP Test** = **((PAPdiff x PAPcost)/18) x 12**

PAPdiff = patient population eligible x the difference in patients screened between intervention and control groups

PAPcost = current cost of a PAP test

## Savings Associated with the Reduction of Inappropriate Preventive Care

**1) CHEST X-Ray = ((CHESTdiff x CHESTcost)/18) x 12**

CHESTdiff = patient population eligible x the difference in patients screened between intervention and control groups

CHESTcost = current cost of a chest x-ray (CXR)

**2) Mammography 40 to 49 = (((MAMMdiff x MAMMcost)/18) x 12)+(MAMMdiff x CANCERinc x BIOPSY)**

MAMMdiff = patient population eligible x the difference in patients screened between intervention and control groups

MAMMcost = current cost of a bilateral mammogram

CANCERinc = incidence of breast cancer

BIOPSY = current cost of biopsy

**3) PSA Testing =** (**(PSAdiff x PSApos x BIOPSY)/18)x12)**

PSAdiff = patient population eligible x the difference in patients screened between intervention and control groups

PSApos = false positive rate for PSA

BIOPSY = current cost of biopsy

**4) Blood Glucose =((BGdiff x BGtest1)/18)x12) +(BGprop1*BGdiff*BGpos*BGtest1)+(BGprop2*BGdiff*BGpos*BGtest2)**

BGdiff = patient population eligible x the difference in patients screened between intervention and control groups

BGtest1 = current cost of a fasting blood glucose test

BGprop1 = estimated proportion of eligible patients for follow-up

BGpos = false positive rate for initial Blood Glucose screen

BGprop2 = estimated proportion of eligible patients for follow-up with glucose tolerance test

BGtest2 = current cost of glucose tolerance test

**5) Urine Protein = ((UPdiff xUPpos x UPcost)/18)x12)**

UPdiff = patient population eligible x the difference in patients screened between intervention and control groups

UPcost = current cost of a urine culture and urineanalysis

UPpos = false positive rate for urine dipstick test

## Savings from the Provision of Appropriate Preventive Care

**1) Breast Cancer = (BCdiff x BCinc x BCtrtcost)**

BCdiff = patient population eligible x the difference in patients screened between intervention and control groups

BCinc = incidence of breast cancer in the eligible population

BCtrtcost = current treatment costs saved for each women screened

**2) Influenza = (FLUdiff x FLUpneu x FLUcost1)+(FLUdiff x FLUresp x FLUcost2) + (FLUdiff x FLUhrt x FLUcost3) + (FLUdiff x FLUemerg x FLUcost4)**

FLUdiff = patient population eligible x the difference in patients vaccinated between intervention and control groups

FLUpneu = pneumonia hospitalizations averted

FLUcost1 = current cost of pneumonia hospitalization

FLUresp = chronic respiratory hospitalizations averted

FLUcost2 = current cost of respiratory hospitalization

FLUhrt = congestive heart failure hospitalizations averted

FLUcost3 = current cost of heart failure hospitalization

FLUemerg = emergency room visits avoided

FLUcost4 = current cost of an emergency room visit

**3) Neural Tube Defects = (FOLICDiff x NTprob x NTcost)**

FOLICdiff = patient population eligible x the difference in patients counselled between intervention and control groups

NTprob = probability of reducing neural tube defects from folic acid intake

NTcost = life-time direct treatment costs for spina bifida

**4) Cervical Cancer = (PAPdiff x CCinc x CCcost)**

PAPdiff = patient population eligible x the difference in patients screened between intervention and control groups

CCinc = incidence of cervical cancer in the eligible patient population

CCcost = current treatment costs saved for each women screened

**5) Lung Cancer = NRTeff x (CESSdiff x LCinc) x LCcost**

CESSdiff = patient population eligible x the difference in patients screened between intervention and control groups

LCinc = incidence of lung cancer in smokers

NRTeff = reported efficacy of nicotine replacement therapy

LCcost = current treatment costs for lung cancer

**6) Heart Disease = (HTdiff x HDinc x HDcost)**

HTdiff = patient population eligible x the difference in patients treated for hypertension between intervention and control groups

HDinc = incidence of heart disease in eligible population

HDcost = current treatment cost for heart disease

**7) STD Treatment = PIDprev x (STDdiff x STDinc) x PIDcost**

STDdiff = patient population eligible x the difference in patients screened between intervention and control groups

PIDprev = pelvic inflammatory disease(PID) prevented

STDinc = incidence of gonorrhoeae and Chlamydia in eligible patient population

PIDcost = current treatment costs for PID

**8) Stroke = (HTdiff x STRKinc x STRKcost)**

HTdiff = patient population eligible x the difference in patients treated for hypertension between intervention and control groups

STRKinc = incidence of stroke in eligible population

STRKcost = current treatment cost for stroke

1. The intervention was 18 months in duration. All screening costs have been converted to a 12 month period where appropriate. [↑](#footnote-ref-2)
